# A plant-based medicinal food inhibits the growth of human gastric carcinoma by reversing epithelial–mesenchymal transition via the canonical Wnt/β-catenin signaling pathway

**DOI:** 10.1186/s12906-021-03301-6

**Published:** 2021-05-08

**Authors:** Xuxi Chen, Wuyang Yue, Lin Tian, Na Li, Yiyi Chen, Lishi Zhang, Jinyao Chen

**Affiliations:** 1 West China School of Public Health and West China fourth Hospital, Sichuan University, Chengdu, China; 2 Food Safety Monitoring and Risk Assessment Key Laboratory of Sichuan Province, Chengdu, China; 3 Department of Tuberculosis Institute Research, Chongqing Public Health Medical Center/Public Health Hospital Affiliated to Southwest University, Chongqing, China

**Keywords:** Gastric cancer, Medicinal food, Epithelial–mesenchymal transition, Wnt/β–catenin signaling pathway

## Abstract

**Background:**

Natural products, especially those with high contents of phytochemicals, are promising alternative medicines owing to their antitumor properties and few side effects. In this study, the effects of a plant-based medicinal food (PBMF) composed of six medicinal and edible plants, namely, *Coix* seed, *Lentinula edodes*, *Asparagus officinalis* L., *Houttuynia cordata*, *Dandelion*, and *Grifola frondosa*, on gastric cancer and the underlying molecular mechanisms were investigated in vivo.

**Methods:**

A subcutaneous xenograft model of gastric cancer was successfully established in nude mice inoculated with SGC-7901 cells. The tumor-bearing mice were separately underwent with particular diets supplemented with three doses of PBMF (43.22, 86.44, and 172.88 g/kg diet) for 30 days. Tumor volumes were recorded. Histopathological changes in and apoptosis of the xenografts were evaluated by hematoxylin and eosin staining and terminal deoxynucleotidyl transferase-mediated dUTP nick end labeling staining, respectively. Serum levels of TNF-α, MMP-2, and MMP-9 were detected by enzyme-linked immunosorbent assay. The mRNA expression levels of *β-catenin*, *GSK-3β*, *E-cadherin*, *N-cadherin*, *MMP-2/9*, *Snail*, *Bax*, *Bcl-2*, *Caspase-3/9*, and *Cyclin D1* were evaluated via real-time quantitative polymerase chain reaction. The protein expression levels of GSK-3β, E-cadherin, N-cadherin, and Ki-67 were determined by immunohistochemistry staining.

**Results:**

PBMF treatment efficiently suppressed neoplastic growth, induced apoptosis, and aggravated necrosis in the xenografts of SGC-7901 cells. PBMF treatment significantly decreased the serum levels of MMP-2 and MMP-9 and significantly increased that of TNF-α. Furthermore, PBMF treatment notably upregulated the mRNA expression levels of *GSK-3β*, *E-cadherin*, *Bax*, *Caspase-3*, and *Caspase-9* but substantially downregulated those of *β-catenin*, *N-cadherin*, *MMP-2*, *MMP-9*, *Snail*, and *Cyclin D1* in tumor tissues. The Bax/Bcl-2 ratio was upregulated at the mRNA level. Moreover, PBMF treatment remarkably increased the protein expression levels of GSK-3β and E-cadherin but notably reduced those of Ki-67 and N-cadherin in tumor tissues.

**Conclusions:**

The PBMF concocted herein exerts anti-gastric cancer activities via epithelial–mesenchymal transition reversal, apoptosis induction, and proliferation inhibition. The underlying molecular mechanisms likely rely on suppressing the Wnt/β-catenin signaling pathway.

**Supplementary Information:**

The online version contains supplementary material available at 10.1186/s12906-021-03301-6.

## Introduction

Gastric carcinoma (GC) is the fifth most prevalent malignancy with approximately 1,033,701 new cases reported annually; moreover, it is the third leading cause of cancer-related mortality, accounting for about 782,685 deaths every year in both sexes worldwide [1]. In 2015, over 403,000 new cases were diagnosed, and about 290,900 people died from GC in China alone, accounting for about 50% of the global morbidity and mortality [2]. The development and progression of classic therapeutic strategies, such as surgery, radiotherapy, and chemotherapy, in combination with neoadjuvant therapy has prolonged the survival of some patients with GC to some extent. However, the overall prognosis of these patients is considerably poor because of late detection of GC, tumor recurrence, and distant metastasis [3]. Moreover, drug resistance and the various side effects of conventional GC treatments necessitate the development of novel therapeutic strategies to improve their efficiency.

Natural products and their derivatives, especially phytochemicals and dietary compounds, obtained from vegetables, fruits, edible fungi, Chinese traditional herbs, and medicinal plants play pivotal roles in the prevention and treatment of various human cancers [4, 5]. Over 60% of the existing first-line anticancer chemotherapeutic drugs in clinical applications is estimated to have been derived from natural products and their derivatives; thus, they are considered promising candidates for complementary and alternative therapeutic strategy for cancer [6]. Moreover, the use of these products in the treatment of advanced cancer stages has attracted increased attention because they are relatively safe, highly effective, have few toxic adverse effects, widely available, and they enhance the body’s natural immune system [7, 8]. Numerous in vivo and in vitro experimental studies have demonstrated that *Coix* seed, *Lentinula edodes*, *Asparagus officinalis* L., *Houttuynia cordata*, *Dandelion*, and *Grifola frondosa* have potent anticancer properties, such as antiproliferation, antimetastasis, and apoptosis induction; moreover, they have functional bioactive constituents involving a wide range of phytochemicals, such as flavonoids, alkaloids, saponins, terpenoids, polysaccharides, and others [9–14].

The ethanolic extracts of *Coix* seed, a famous Chinese traditional herb, reportedly remarkably inhibit the viability of GC AGS cells [9]. Furthermore, injection of kanglaite, an ingredient extracted from *Coix* seed, has been clinically utilized as a broad-spectrum antitumor drug against GC [15]. *L. edodes* extracts, such as Latcripin 1, can effectively induce apoptosis and suppress metastasis in SGC-7901 and BGC-823 human GC cells [10]. Extracts of *A. offiicinalis* L*.* have a mild antiproliferative activity against human endometrial carcinoma Ishikawa cells [11]. Aerial stem extracts of *H. cordata* Thunb. reportedly exert anticancer effects on GC by inducing apoptosis in SGC-7901 cells [12]. *Dandelion* root extracts can specifically suppress the proliferation and migration of SGC-7901 and BGC-823 GC cells [13]. *G. frondosa* polysaccharides can induce apoptosis in MCF-7 human breast cancer cells and inhibit tumor growth in nude mice xenografts [14]. These pieces of evidence suggest that the combination of these plants may have high efficacy against GC.

A previous study demonstrated that the combination of various phytochemicals can possibly improve their effectiveness owing to their multiple actions on several key targets aimed at tumorigenesis. The same study indicated that such a combination may prevent drug resistance arising from chemotherapeutic drugs that have a single target [16]. Similarly, Andrea et al. [17] reckoned that consumption of plant-derived functional foods mixing various phytochemicals with multiple biological activities appears to be a more logical strategy for the prevention and treatment of cancer than using a single phytochemical [17]. On the basis of these premises, we speculated that a combination of the aforementioned medicinal and edible plants may have anti-GC effects. However, little is known about this aspect, and the underlying mechanisms of action must be investigated.

Invasiveness and metastasis are the primary reasons responsible for high cancer recurrence and mortality rate [18]. Changes in tumor cell adhesion and motility are crucial to the development of invasiveness and metastasis of malignancies [19]. Epithelial–mesenchymal transition (EMT) is regarded as the key element of this process [20, 21]. Recent studies reported that aberrant activation of EMT enables gastric epithelial cell with loss of close adherence to neighboring cells and apical–basal polarity to stepwise transition toward mesenchymal cells. These processes eventually lead to the acquisition of tumorigenic phenotype characteristics of migratory and invasive properties. This phenomenon further establishes that EMT is definitely associated with gastric carcinogenesis. A regulatory series of cellular hallmarks that predominantly include E-cadherin (an epithelial cell biomarker) and N-cadherin (a mesenchymal cell biomarker) have also been validated as early diagnostic markers for GC [22]. The Wnt signaling pathway, a versatile pathway with multiple physiological and pathological functions, can facilitate EMT program with the help of two key regulators, namely, GSK-3β and β-catenin [23]. Furthermore, dysfunction of this pathway has a major effect on gastric tumorigenesis [24].

In this study, we aimed to investigate the therapeutic effects of a plant-based medicinal food (PBMF) developed from the aforementioned medicinal and edible plants on GC by using a subcutaneous xenograft model in nude mice from SGC-7901 cells and explore the underlying mechanisms. Results showed that the underlying mechanisms were principally attributed to EMT reversal via blocking the Wnt/β-catenin signaling pathway.

## Materials and methods

### Test substances and reagents

The PBMF concocted herein, which was a mixture of extracts derived from *Coix* seed, *L. edodes*, *A. officinalis* L., *H. cordata*, *Dandelion*, and *G. frondosa*, was prepared by Jiangxi Gongqin Jiangzhong Dietary Therapy Technology Company (Jiujiang, China).

5-Fluorouracil (5-Fu) (Sigma Aldrich, USA) was dissolved in PBS to the terminal concentration of 2 mg/mL. The prepared 5-Fu solution was sterilized through a 0.22 μm-thick filter for subsequent experiments and stored at 4 °C.

Antibodies against GSK-3β, E-cadherin, N-cadherin, and Ki-67 were purchased from Servicebio Technology Company (Wuhan, China). Anti-rabbit and anti-mouse secondary horseradish peroxidase (HRP) antibodies were also bought from Servicebio Technology Company (Wuhan, China). Enzyme-linked immunosorbent assay (ELISA) was performed by Mouse Premixed Multi-Analyte Kit (R&D Systems, USA). Terminal deoxynucleotidyl transferase-mediated dUTP nick end labeling (TUNEL) staining was conducted by In Situ Cell Death Detection Kit (fluorescein) (Roche Applied Science, Germany). Animal Total RNA Isolation Kit was acquired from Foregene Biotechnology Company (Chengdu, China) to extract RNA. Iscript cDNA Synthesis Kit and SsoFast EvaGreen Supermix Kit were obtained from Bio-Rad (USA) to carry out real-time quantitative polymerase chain reaction (RT-qPCR). The other reagents used were all commercially available.

### Cell lines and cell culture

SGC-7901, a human GC cell line, was obtained from Hali Biotechnology (Chengdu, China). The cells were cultured in PRMI-1640 supplemented with 10% (v/v) fetal bovine serum (Gibco, USA) and 1% (v/v) penicillin/streptomycin (Hyclone, USA). The cultures were incubated in the humidified atmosphere of 5% CO_2_ at 37 °C.

### Animal

Thirty male nude mice (BALB/c nu/nu, SPF level, 4–6 weeks old, approximately 14–16 g in weight) were purchased from Charles River Laboratory Animal Technology Company (Beijing, China) (Permit No.: SYXK [jing] 2016–0006). All mice were raised under a specific pathogen-free environment (25 °C ± 2 °C, 50% ± 10% humidity) with a 12/12 h light/dark cycle in the Laboratory Animal Center of West China Second University Hospital (Permit No.: SYXK [chuan] 2018–209). Procedures involving animal experiments and care were implemented in accordance with the Guide for the Care and Use of Laboratory Animals of Sichuan University and were approved by the Animal Ethics Committee of our Institute (Animal ethical approval No.: 2019–068). All mice were fed with standard laboratory pellet diet and water ad libitum for 1 week to acclimate prior to the conduct of the experiments.

### Establishment of subcutaneous xenograft tumor model and experimental design

SGC-7901 cells (2 × 10^6^) were resuspended in 200 μL of serum- and antibiotic-free PRIM 1640 and injected subcutaneously into the right armpit of all nude mice. After 3 weeks of growth, the xenograft tumors caused by the SGC-7901 cells in each group were all palpable and reached about 3–4 mm in diameter on average, indicating that the subcutaneous xenograft tumor model was successfully established. Subsequently, the tumor-bearing mice were randomly divided into five groups (*n* = 6 each group): model control group (0.2 mL PBS by intraperitoneal injection and normal diet), 5-Fu group (0.2 mL 5-Fu [20 mg/kg] by intraperitoneal injection and normal diet), low-dose group (0.2 mL PBS by intraperitoneal injection and a particular diet containing the PBMF at a concentration of 43.22 g/kg diet), medium-dose group (0.2 mL PBS by intraperitoneal injection and a particular diet containing the PBMF at a concentration of 86.44 g/kg diet), and a high-dose group (0.2 mL PBS by intraperitoneal injection and a particular diet containing the PBMF at a concentration of 172.88 g/kg diet).

Treatment was started on the same day of initial randomization. The tumor-bearing mice in the PBMF-treated groups were fed with the three particular diets, except for the model control group and the 5-Fu group, which were fed with normal diet. PBS and 5-Fu were intraperitoneally administered every other day for the first 2 weeks. The entire experimental period lasted for 30 days. Body weights were recorded, and tumor volumes were measured by Vernier calipers twice every week. Tumor size was calculated using the formula *TV* = length (mm) × width^2^ (mm^2^) × 0.5.

All mice were euthanized by cervical dislocation after fasting overnight at the end of the experiments. The tumors were harvested and weighed. The inhibition rate (IR) of tumor growth was computed using the formula *IR* = (1 − *W*_*T*_/*W*_*C*_) × 100%, where *W*_*T*_ is the mean tumor weight of the treatment groups, and *W*_*C*_ is the mean tumor weight of the model control group.

Portions of the tumor tissues were fixed in 4% paraformaldehyde solution. The portions were embedded in paraffin, sectioned, and then subjected to hematoxylin and eosin (H&E) staining, immunohistochemistry analysis, and terminal deoxynucleotidyl transferase-mediated dUTP nick end labeling (TUNEL) assay. Another portion was immediately stored at − 80 °C for subsequent RNA extraction.

### ELISA of TNF-α, MMP-2, and MMP-9 levels in serum

Blood samples of the tumor-bearing mice were collected from abdominal aorta. After standing at room temperature for 2 h, serum was isolated from the blood samples by centrifugation at 3000 rpm for 15 min at 4 °C. The resulting supernatants were transferred into microcentrifuge tubes and stored immediately at − 80 °C until analysis. The serum levels of tumor necrosis factor-α (TNF-α), matrix metalloproteinase-2 (MMP-2), and matrix metalloproteinase-9 (MMP-9) were determined via ELISA on Luminex 200 following the manufacturer’s instructions (R&D Systems, USA).

### Histopathological examination of tumor tissues

Fresh xenograft tissues from all five groups were fixed at 4% paraformaldehyde solution for 48 h before they were embedded in paraffin. The paraffin-embedded blocks were then cut into 4 μm-thick sections for further histopathological examination, followed by routine H&E staining. The histomorphology of the tumor cells was observed at 200× magnification in two randomly selected nonrepetitive fields on each slide under an optical microscope (Olympus, Tokyo, Japan, BX53).

### TUNEL staining

The apoptosis of the xenografts caused by the SGC-7901 cells were measured via TUNEL staing by using an In Situ Cell Death Detection Kit (fluorescein; Roche Applied Science, Germany). Three representative paraformaldehyde-fixed and paraffin-embedded tumor samples were collected from each group. Serial 4 μm-thick sections were cut from these samples for subsequent TUNEL staining and conducted in accordance with the manufacturer’s instructions. Tumor cells with green-fluorescence nuclei were regarded as TUNEL positive. Total cells were counterstained with DAPI. The proportion of positive cells was estimated at high magnifications in five randomly selected nonrepetitive fields on each slide under a fluorescence microscope (Leica, Germany, DM4000B). The percentage of TUNEL-positive cells reflecting the apoptosis status was calculated using the formula TUNEL positive rate = positive cells (green-fluorescence nuclei)/total cells (blue-fluorescence nuclei) × 100%.

### RT-qPCR of *β-catenin*, *GSK-3β*, *E-cadherin*, *N-cadherin*, *MMP-2*, *MMP-9*, *Snail*, *Bcl-2*, *Bax*, *Caspase-3*, *Caspase-9*, and *Cyclin D1* genes in the xenograft tumors

Three representative xenograft tumor samples were collected from each group. Total RNA was extracted from the samples by using Animal Total RNA Isolation Kit (Foregene, China) following the manufacturer’s protocols. RNA concentration was detected by NanoDrop TM 2000 (Thermo, MA, USA), and RNA quality was estimated by OD_260_/OD_280_ (1.9–2.1). The RNA concentration of each sample was quantified to the same level of 500 ng/μL and reverse-transcribed to cDNA by using Iscript cDNA Synthesis Kit (Bio-Rad, USA) according to the manufacturer’s protocols. The sequences of gene-specific primers were commercially synthesized by Sangong Biotech Company (Table 1). Target genes were amplified using cDNA (1 μL), SsoFast EvaGreen Supermix (5 μL, Bio-Rad, USA), forward primer (0.3 μL), reverse primer (0.3 μL), and RNase-free H_2_O up to 10 μL in a CFX96 Real-Time PCR Detection System (Bio-Rad) under the following conditions: enzyme activation at 98 °C for 2 min, followed by 40 cycles of denaturation at 98 °C for 5 s, and annealing/extension at 60 °C for 5 s. The melting curve at 55 °C–60 °C for 5 s was routinely established to confirm the specificity of the primers. Relative gene expression was expressed as *△Cq* = *Cq* (target gene) − *Cq* (reference gene). The *GAPDH* gene was used as an internal control, and its relative mRNA expression was calculated as *△△Cq* = *△Cq* (treatment group) − *△Cq* (model control group). Finally, the relative mRNA expression levels of the target genes were analyzed via the 2^-△△Cq^ method, and the results were presented as fold difference [25].

**Table 1:** Primer sequences used for RT-PCR (5′-3′)

Gene	Sequences
*β-catenin*	Forward: GGTGGACCCCAAGCCTTAGTA
	Reverse: AGATGAAGCCCCAGTGCCT
*GSK-3β*	Forward: CCACCATCCTTATCCCTCCA
	Reverse: AGCGGCGTTATTGGTCTGTC
*E-cadherin*	Forward: CATCGCCTACACCATCGTCA
	Reverse: ACTCTCTCGGTCCAGCCCA
*N-cadherin*	Forward: CGTGGGAATCAGACGGCTA
	Reverse: AAGAGGGAGTCATACGGTGGC
*MMP-2*	Forward: CTCCCCCGATGCTGATACTG
	Reverse: GGTGTCACTGTCCGCCAAAT
*MMP-9*	Forward: CCAAAGACCTGAAAACCTCCAA
	Reverse: TGAAGCATCAGCAAAGCCG
*Snail*	Forward: TTCACCTTCCAGCAGCCCT
	Reverse: TTGCCACTGTCCTCATCGG
*Bcl-2*	Forward: CCCTGGCATCTTCTCCTTCC
	Reverse: TCACGACGGTAGCGACGAG
*Bax*	Forward: CCCGAGAGGTCTTCTTCCG
	Reverse: AAGTCCAGTGTCCAGCCCAT
*Caspase-3*	Forward: CTGACTGGAAAGCCGAAACTC
	Reverse: GGACTGGATGAACCACGACC
*Caspase-9*	Forward: CTTTGATGGAGATGGCACACC
	Reverse: GTCTTTCTGCTCACCACCGC
*Cyclin D1*	Forward: GGAGCAGAAGTGCGAAGAGG
	Reverse: TCACCAGAAGCAGTTCCATTTG
*GAPDH*	Forward: CCTTCCGTGTTCCTACCCC
	Reverse: GCCCAAGATGCCCTTCAGT

### Immunohistochemistry staining of GSK-3β, E-cadherin, N-cadherin, and Ki-67 in the xenograft tumors

All xenografts were harvested from the tumor-bearing mice, fixed at 4% paraformaldehyde for 48 h, and embedded in paraffin. Three representative samples from each group were selected for immunohistochemistry staining. The selected tumor sections were routinely deparaffinized and hydrated. Antigens were retrieved by heating the sections in a buffer (EDTA, pH 9.0; sodium citrate, pH 6.0) in a microwave oven for 15 min. The sections were incubated in 3% H_2_O_2_ at room temperature and away from light for 15 min. The sections were blocked with 5% BSA at room temperature for 30 min and incubated overnight with primary antibodies specific for GSK-3β (1:500 dilution; Servicebio), E-cadherin (1:800 dilution; Servicebio), N-cadherin (1:200 dilution; Servicebio), and Ki-67 (12,000 dilution; Servicebio) at 4 °C. The next day, the sections were incubated with HRP-conjugated secondary antibodies (Servicebio) at room temperature for 50 min after washing in PBS for three times (5 min each time). Target protein expression was detected by coloration with 3′-diaminobenzidine (DAB) buffer (Servicebio), and the sections were counterstained with hematoxylin (Servicebio) for 3 min. All of the images were visualized under microscope (CIC, XSP-C204). Pale brown tumor cells were regarded as positive cells. GSK-3β-positive staining was notable in the cytoplasm and nucleus, while E-cadherin- and N-cadherin-positive staining were evident in the cytomembrane and cytoplasm. Ki-67-positive staining was notable in the nucleus. Image-pro Plus 6.0 software was used to calculate the average integrated optical density (IOD) per stained area (IOD/area) for positive staining.

### Statistical analysis

All data was expressed as mean ± standard deviation (SD) of at least three independent experiments and analyzed using one-way analysis of variance (ANOVA) by SPSS 21.0 software. Comparison among different groups was performed using LSD test. A *p*-value of less than 0.05 was considered as a statistically significant different.

## Results

### Body weight and general condition of nude mice

**Fig. 1:**
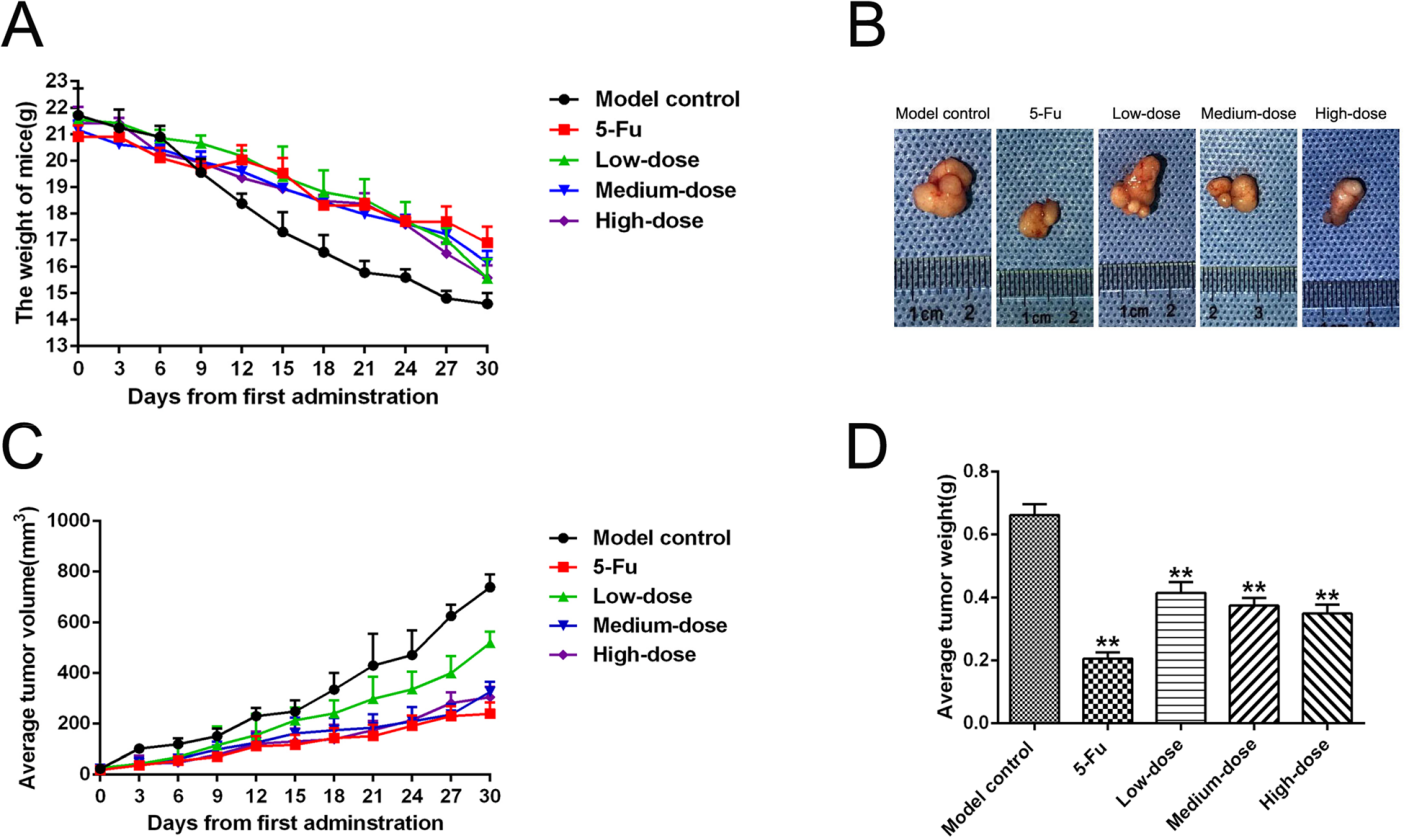
PBMF inhibited SGC-7901 xenograft tumors growth in nude mice. **a** Dynamic changes of tumor weights in each group during different treatments for 30 days as designed. Tumor weights were recorded once every 3 day. **b** Representative photographs of tumors isolated from mice in each group after sacrifice. **c** Dynamic changes of tumor volumes in each group during entire experiment. Tumor volumes were measured by vernier calipers and recorded once every 3 day. **d** All tumors were weighed on day 30. Tumor weights of 5-Fu group as well as low-, medium-dose and high-dose group are significantly less than that of model control group. Results are represented as mean weight ± SD. ^**^p < 0.01 vs. model control. (Each part of the multi-panel Fig. 1 see Additional file 1)

A subcutaneous xenograft tumor model from SGC-7901 cells was successfully established in 30 nude mice by using the aforementioned experimental protocol. In the final stage of the experiment, the activity and adaptive response to exogenous stimulus decreased in each group of tumor-bearing mice. As shown in the Fig. 1a and Table 2, the body weight among each group unevenly declined, and the loss of body weight in the model control group was severe. The terminal loss of body weight significantly decreased in 5-Fu and PBMF-treated groups compared with that in the model control group (*p* < 0.01).

**Table 2:** Body weights of nude mice bearing SGC-7901 xenografts (^−^*x* ± *s*, *n* = *6*)

Group	Body weights at day 0 (g)	Body weights at day 30 (g)	The terminal loss of body weights^a^ (g)
Model control	22.73 ± 0.14	14.60 ± 0.41	8.13 ± 0.27
5-Fu	20.91 ± 0.43	16.89 ± 0.62	4.02 ± 0.99^**^
Low-dose	21.57 ± 0.24	15.57 ± 0.73	6.00 ± 0.86^**^
Medium-dose	21.17 ± 0.36	16.16 ± 0.44	5.01 ± 0.40^**^
High-dose	21.44 ± 0.61	15.59 ± 0.47	5.85 ± 0.28^**^

^**^p < 0.01 vs. model control

^a^ The terminal loss values of body weights = body weights at day 0 - body weights at day 30

### Tumor growth in SGC-7901 xenograft tumor model

At the end of the experiment, all tumor-bearing mice were euthanized, and tumors were harvested and weighed (Fig. 1b). Treatment with 5-Fu and low, medium, and high doses of PBMF significantly decreased the tumor weight compared with that in the model control group (Fig. 1d and Table 3). As shown in Fig. 1c and Tables 3, 5-Fu as well as low-, medium-, and high-dose PBMF significantly inhibited the growth of xenograft tumor by 68.90, 37.27, 43.41, and 47.23%, respectively, compared with that in the model control group.

**Table 3:** Effects of PBMF on the growth of SGC-7901 xenograft tumors (^−^*x* ± *s*, *n* = *6*)

Group	Tumor volume^a^ (mm^3^)	Tumor weight^a^ (g)	The inhibition rates of tumor growth (%)
Model control	734.49 ± 49.97	0.662 ± 0.03	–
5-Fu	239.52 ± 44.82^**^	0.206 ± 0.02^**^	68.90
Low-dose	519.52 ± 44.50^**^	0.416 ± 0.03^**^	37.27
Medium-dose	326.45 ± 39.57^**^	0.375 ± 0.02^**^	43.41
High-dose	305.59 ± 39.81^**^	0.350 ± 0.03^**^	47.23

^**^p < 0.01 vs. model control

^a^ Tumor volume and weights were measured after all nude mice were sacrificed

### Histopathological determination

**Fig. 2:**
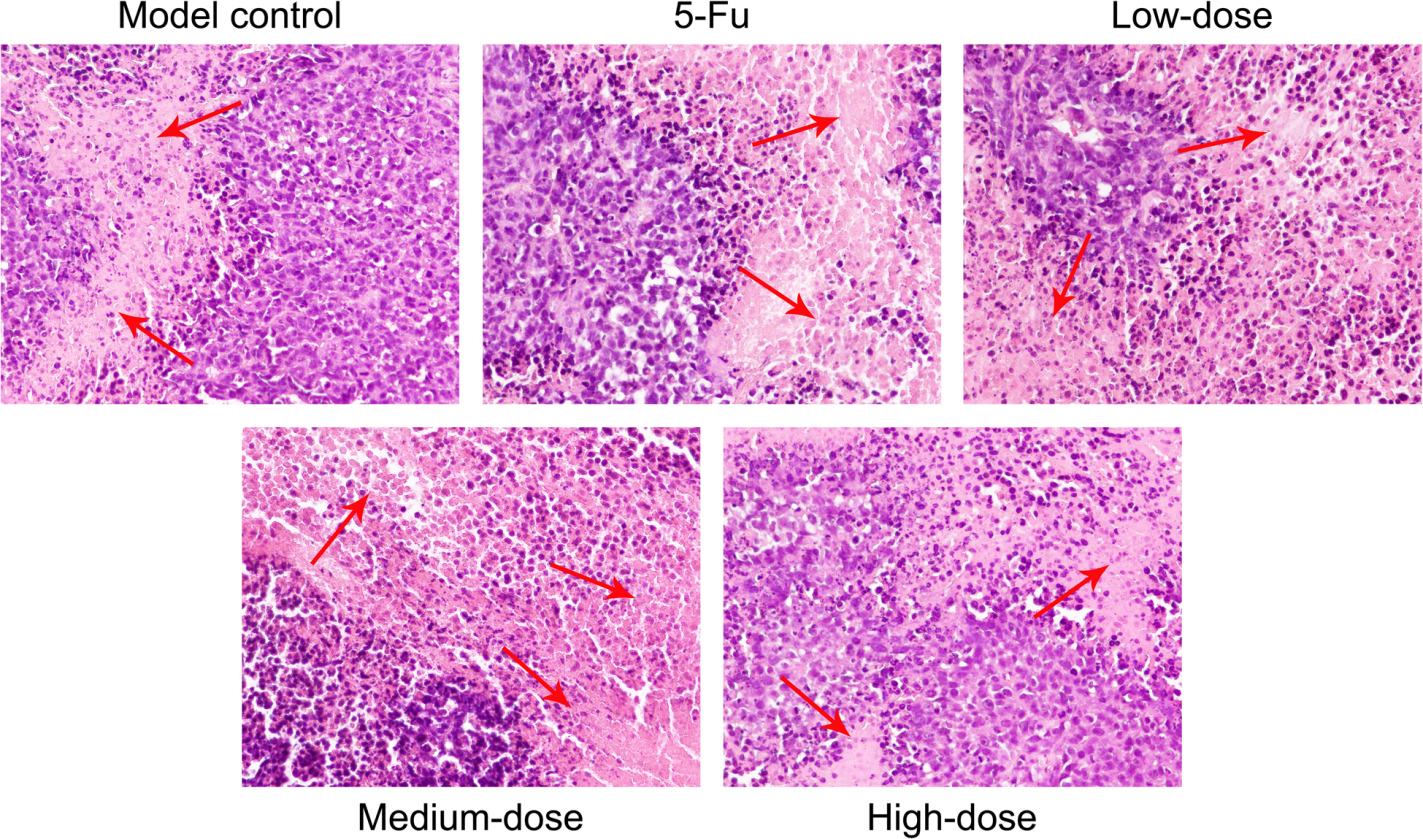
Histopathological changes of SGC-7901 xenograft tumor tissues. Tumor necrosis areas were determined by H&E staining and observed under light microscope (200×). Red arrows refer to the necrosis areas. (Each part of the multi-panel Fig. 2 see Additional file 2)

Based on H&E staining, necrosis was evident in the tumor tissues in 5-Fu and PBMF-treated groups and was more severe than that in the model control group. Necrosis in the medium-dose group was the most pronounced, and its area of necrosis accounted approximately for 2/3 of the total area under light microscope (Fig. 2).

### Tumor cells apoptosis

**Fig. 3:**
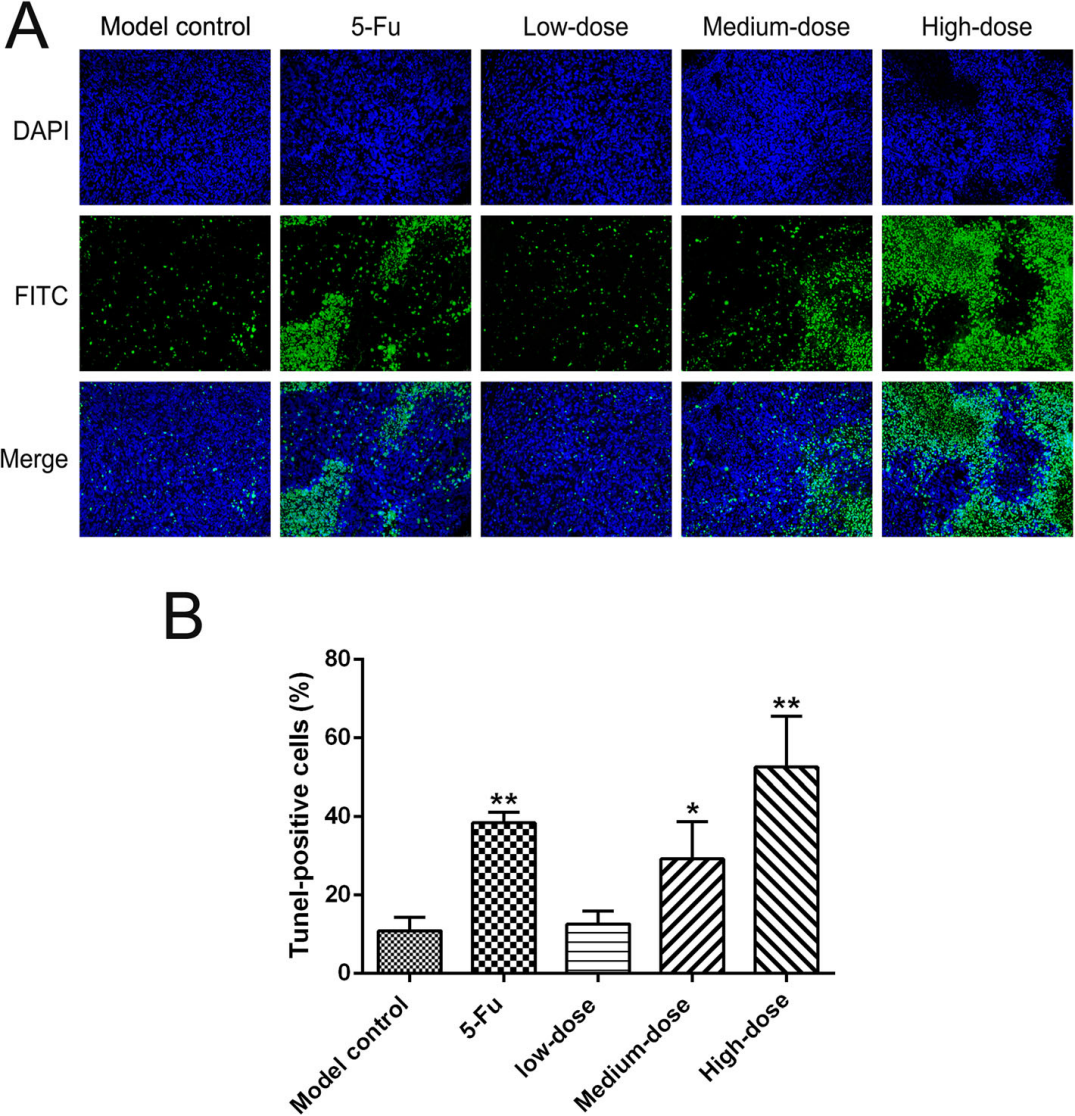
Effects of PBMF on apoptosis induction of tumor cells in SGC-7901 xenograft tumor model. **a** TUNEL staining of xenograft tumor tissues was performed and observed under fluorescent microscope (200×). Nuclei with green fluorescence were considered as TUNEL positive cells, while nuclei with blue fluorescence were consider as total cancer cells. **b** Number of TUNEL positive cells was calculated at high magnification in five random fields under a fluorescent microscope. Results were represented as mean ± SD. ^*^p < 0.05 vs. model control; ^**^p < 0.01 vs. model control. (Each part of the multi-panel Fig. 3 see Additional file 3)

TUNEL staining was conducted in xenograft tumor tissues to evaluate the effects of PBMF on apoptosis induction. As shown in Fig. 3, the percentage of TUNEL-positive cells of tumor tissues in the model control group was 10.90% ± 3.39%. Meanwhile, the TUNEL positive rates in 5-Fu, medium-, and high-dose groups were 38.43% ± 2.76, 29.26% ± 9.44, and 52.55% ± 12.94%, respectively, which were all significantly higher than that in the model control group (*p* < 0.05 or *p* < 0.01). Furthermore, the TUNEL positive rate in the low-dose group was 12.65% ± 3.30%, which was slightly higher than that in the model control group but without statistical significance (*p* > 0.05).

### Serum TNF-α, MMP-2, and MMP-9 levels

**Fig. 4:**
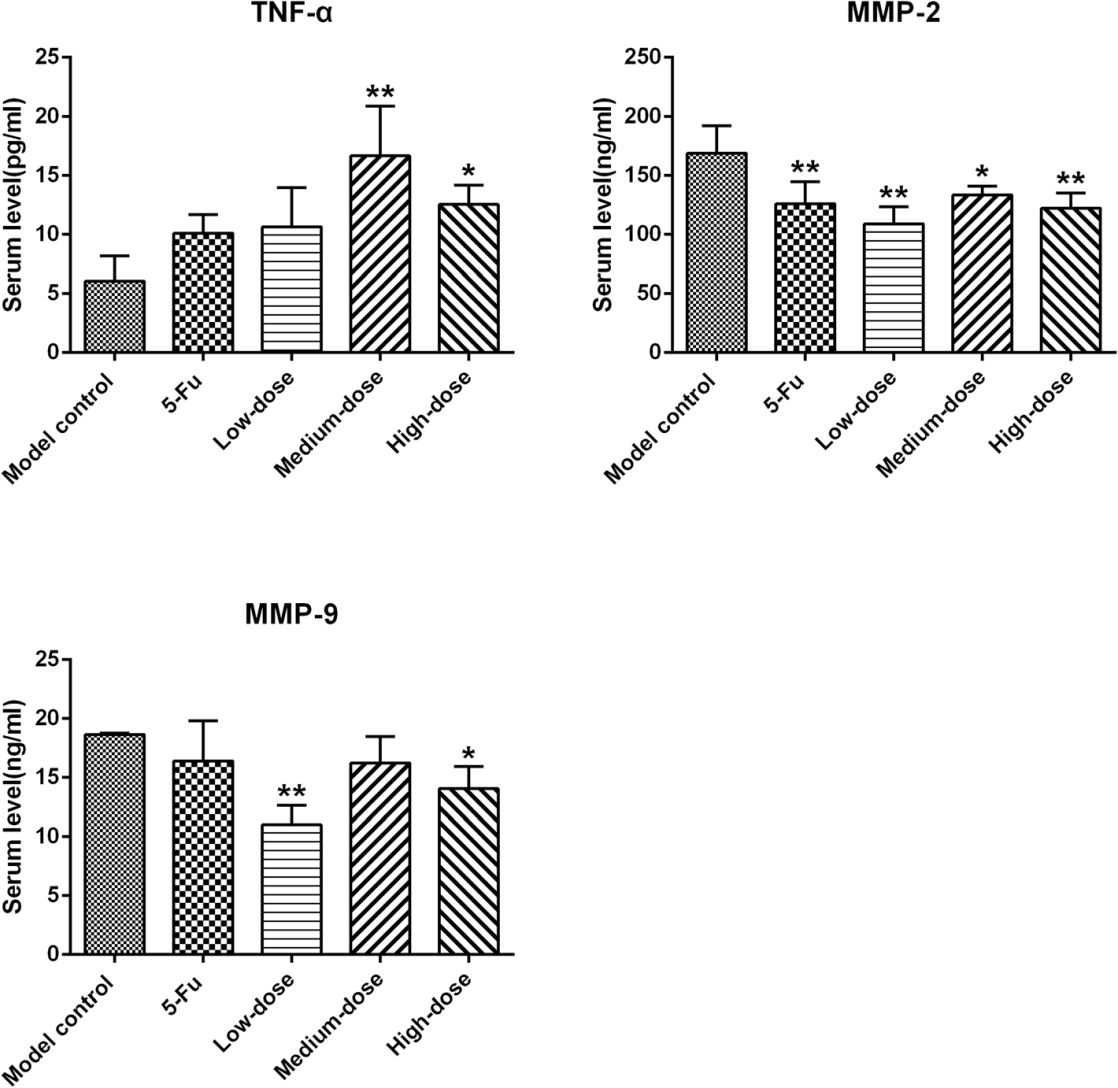
Effects of PBMF on serum levels of TNF-α, MMP-2 and MMP-9 in nude mice. Results were represented as mean ± SD. ^*^p < 0.05 vs. model control group; ^**^p < 0.01 vs. model control. (Each part of the multi-panel Fig. 4 see Additional file 4)

As shown in Table 4 and Fig. 4, the serum level of TNF-α in medium- and high-dose groups was significantly elevated compared with that in the model control group (*p* < 0.05 or *p* < 0.01). However, the serum level of MMP-2 in the 5-Fu group as well as PBMF-treated groups was significantly lower than that in the model control group (*p* < 0.05 or *p* < 0.01). The serum level of MMP-9 in low- and high-dose groups was significantly lower than that in the model control group (*p* < 0.05 or *p* < 0.01).

**Table 4:** Serum levels of TNF-α, MMP-2 and MMP-9 in nude mice (^−^*x* ± *s*, *n* = 3)

Group	TNF-α (pg/mL)	MMP-2(ng/mL)	MMP-9 (ng/ml)
Model control	6.07 ± 2.13	169.08 ± 23.07	18.66 ± 0.10
5-Fu	10.12 ± 1.57	125.92 ± 19.00^**^	16.40 ± 3.40
Low-dose	10.67 ± 3.32	108.93 ± 14.54^**^	11.01 ± 1.65^**^
Medium-dose	16.68 ± 4.21^**^	133.48 ± 7.72^*^	16.24 ± 2.22
High-dose	12.56 ± 1.64^*^	122.31 ± 12.72^**^	14.05 ± 1.88^*^

^*^p < 0.05 vs. model control; ^**^p < 0.01 vs. model control

### mRNA expression of specific genes involved in EMT and Wnt/β-catenin signaling pathway in SGC-7901 xenograft tumors

**Fig. 5:**
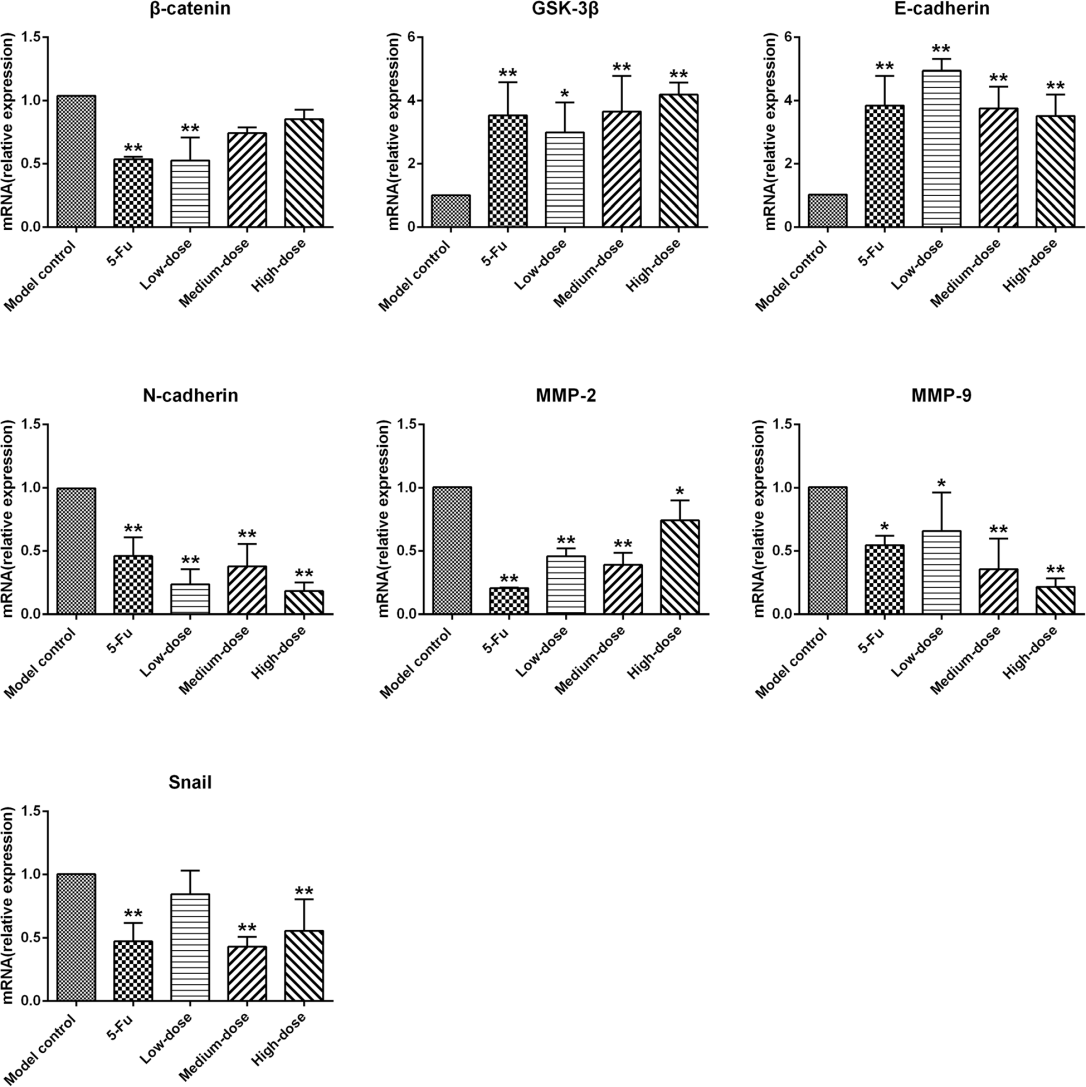
RT-PCR showing the effects of PBMF on mRNA expression involved in EMT and Wnt/β-catenin signaling pathway in tumor tissues. Results were represented as mean ± SD. ^**^p < 0.01 vs. model control. (Each part of the multi-panel Fig. 5 see Additional file 5)

RT-qPCR was applied to determine the effect of PBMF on EMT reversal mediated by the Wnt/β-catenin signaling pathway in xenograft tumor tissues. As shown in Table 5 and Fig. 5, treatment with 5-Fu and three doses of PBMF significantly up-regulated the mRNA levels of *GSK-3β* and *E-cadherin* (*p* < 0.05 or *p* < 0.01). By contrast, the mRNA levels of *N-cadherin*, *MMP-2*, and *MMP-9* in 5-Fu group as well as PBMF-treated groups were significantly lower than those in the model control group (*p* < 0.05 or *p* < 0.01). Compared with those in the model control group, the mRNA level of *β-catenin* was significantly down-regulated in 5-Fu and low-dose PBMF groups, but that of *Snail* significantly decreased in 5-Fu group as well as medium- and high-dose PBMF groups (*p* < 0.05 or *p* < 0.01).

**Table 5:** Relative mRNA levels of specific genes involved in EMT and Wnt/β-catenin signaling pathway in SGC-7901 xenograft tumor tissues (^−^*x* ± *s*, *n* = 3)

Group	*β-catenin*	*GSK-3β*	*E-cadherin*	*N-cadherin*	*MMP-2*	*MMP-9*	*Snail*
Model control	1	1	1	1	1	1	1
5-Fu	0.54 ± 0.02^**^	3.53 ± 1.05^**^	3.85 ± 0.94^**^	0.46 ± 0.15^**^	0.21 ± 0.01^**^	0.55 ± 0.08^*^	0.47 ± 0.15^**^
Low-dose	0.53 ± 0.18^**^	3.00 ± 0.95^*^	4.95 ± 0.37^**^	0.24 ± 0.12^**^	0.46 ± 0.06^**^	0.66 ± 0.30^*^	0.85 ± 0.19
Medium-dose	0.74 ± 0.05	3.66 ± 1.13^**^	3.76 ± 0.69^**^	0.38 ± 0.18^**^	0.39 ± 0.10^**^	0.36 ± 0.24^**^	0.43 ± 0.08^**^
High-dose	0.85 ± 0.08	4.19 ± 0.39^**^	3.52 ± 0.66^**^	0.18 ± 0.07^**^	0.74 ± 0.16^*^	0.22 ± 0.07^**^	0.56 ± 0.25^**^

Data are expressed as fold change. ^*^p < 0.05 vs. model control; ^**^p < 0.01 vs. model control

### mRNA expression of specific genes associated with apoptosis and proliferation in SGC-7901 xenograft tumors

**Fig. 6:**
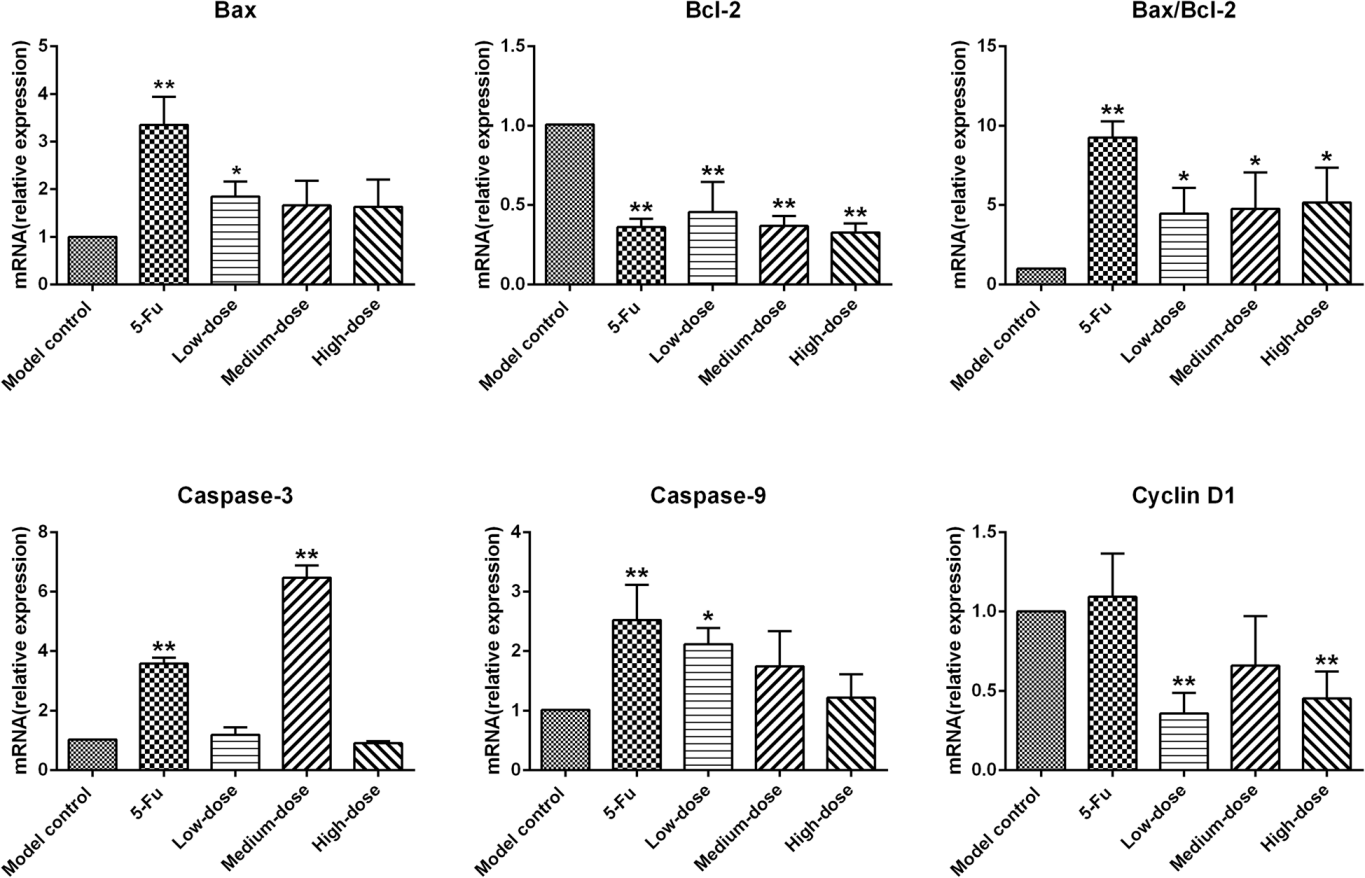
RT-PCR showing the effects of PBMF on mRNA expression involved in apoptosis and proliferation in tumor tissues. Results were represented as mean ± SD. ^*^p < 0.05 vs. model control group; ^**^p < 0.01 vs. model control group. (Each part of the multi-panel Fig. 6 see Additional file 6)

In this study, we further investigated the potential mechanism through which PBMF may induce apoptosis and suppress proliferation in xenograft tumors by RT-PCR. As shown in Table 6 and Fig. 6, the mRNA expression levels of *Bax* and *Caspase-9* were significantly up-regulated in 5-Fu and low-dose PBMF groups, but the mRNA expression of *Caspase-3* was significantly elevated in 5-Fu and medium-dose PBMF groups (*p* < 0.05 or *p* < 0.01). The results also indicated less significant mRNA expression of *Bcl-2* in 5-Fu group as well as PBMF-treated groups than that in the model control group (*p* < 0.01). The mRNA expression of *Cyclin D1* in low- and high-dose PBMF groups was significantly lower than that in the model control group (*p* < 0.01). Furthermore, the Bax/Bcl-2 ratio markedly increased in 5-Fu group as well as PBMF-treated groups compared with that in the model control group (*p* < 0.05 or *p* < 0.01).

**Table 6:** Relative mRNA levels of specific genes involved in apoptosis and proliferation in SGC-7901 xenograft tumor tissues (^−^*x* ± *s*, *n* = 3)

Group	*Bax*	*Bcl-2*	*Bax/Bcl-2*	*Caspase-3*	*Caspase-9*	*Cyclin D1*
Model control	1	1	1	1	1	1
5-Fu	3.36 ± 0.59^**^	0.36 ± 0.05^**^	9.27 ± 1.01^**^	3.60 ± 0.18^**^	2.53 ± 0.59^**^	1.09 ± 0.27
Low-dose	1.84 ± 0.32^*^	0.46 ± 0.19^**^	4.47 ± 1.61^*^	1.19 ± 0.25	2.12 ± 0.27^*^	0.36 ± 0.13^**^
Medium-dose	1.66 ± 0.52	0.37 ± 0.06^**^	4.77 ± 2.30^*^	6.47 ± 0.42^**^	1.75 ± 0.59	0.66 ± 0.31
High-dose	1.63 ± 0.57	0.33 ± 0.06^**^	5.16 ± 2.19^*^	0.91 ± 0.08	1.22 ± 0.40	0.45 ± 0.17^**^

Data are expressed as fold change. ^*^p < 0.05 vs. model control; ^**^p < 0.01 vs. model control

### Expression of representative proteins associated with EMT, Wnt/β-catenin signaling pathway, and cell proliferation in SGC-7901 xenograft tumor tissues

**Fig. 7:**
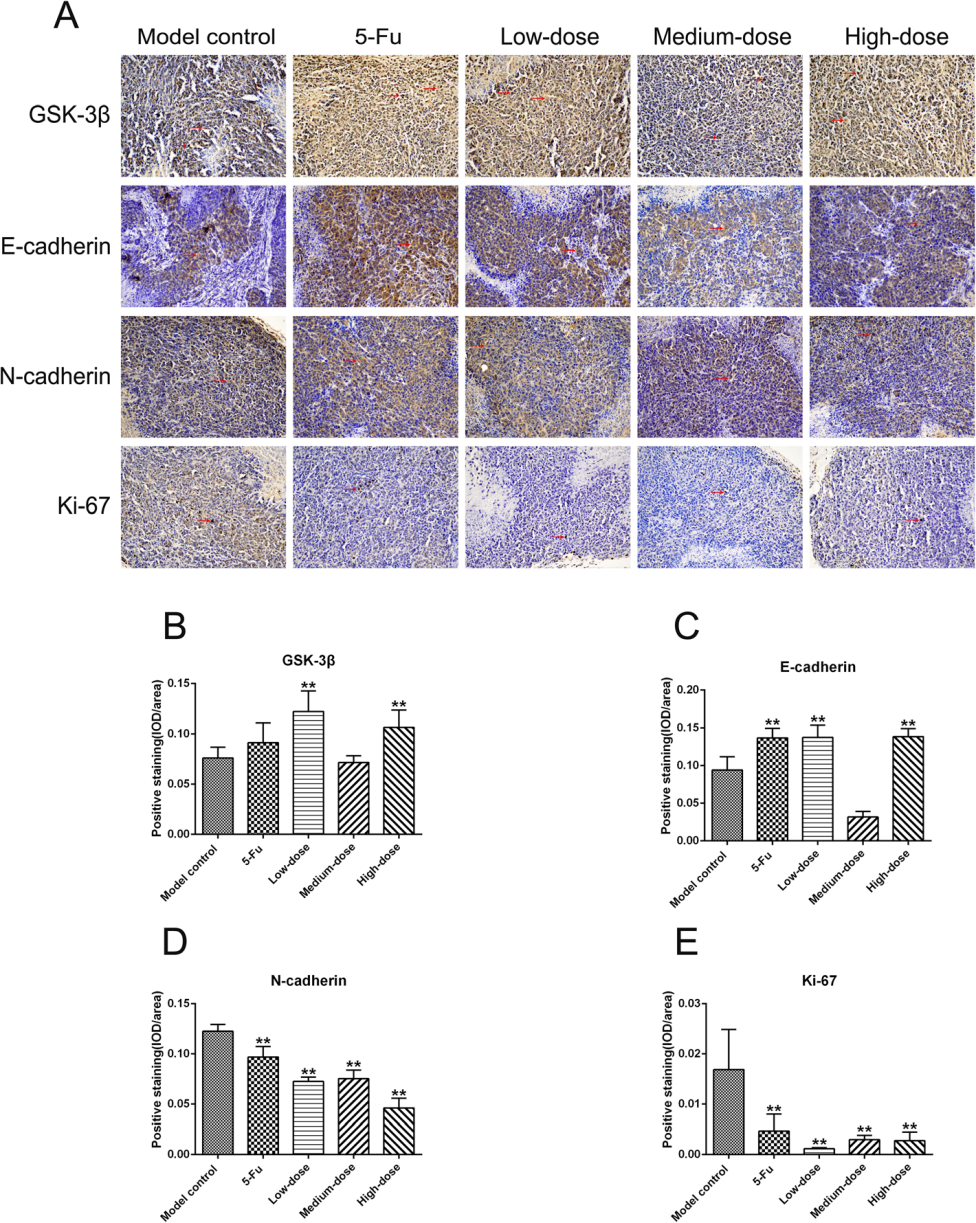
Effects of PBMF on protein expressions of GSK-3β, E-cadherin, N-cadherin and Ki-67 in tumor tissues. **a** Immunohistochemistry staining of GSK-3β, E-cadherin, N-cadherin and Ki-67 proteins among each group was performed and observed under light microscope (200×). GSK-3β proteins were markedly expressed in the cytoplasm and nucleus; E-cadherin and N-cadherin proteins were markedly expressed in cytomembrane and cytoplasm; Ki-67 proteins were markedly expressed in the nucleus. Red arrows refer to positive staining with pale brown. Semi-quantitative analysis of the intensity of GSK-3β-positive (**b**), E-cadherin-positive (**c**), N-cadherin-positive (**d**) and Ki-67-positive (**e**) staining was conducted using IOD/area value. ^**^p < 0.01 vs. model control. (Each part of the multi-panel Fig. 7 see Additional file 7)

Immunohistochemistry staining was performed in xenograft tissues to determine the effects of PBMF on the protein levels of GSK-3β, E-cadherin, N-cadherin, and Ki-67 and further validate its potential molecular mechanisms. As shown in Fig. 7a and b, the expression of GSK-3β in low- and high-dose PBMF groups was significantly up-regulated compared with that in the model control group (*p* < 0.01). The expression of GSK-3β in the 5-Fu group was slightly higher than that in the model control group, but the difference was not statistically significant (*p* > 0.05). The expression of E-cadherin in 5-Fu group as well as low- and high-dose PBMF groups was significantly up-regulated compared with that in the model control group (*p* < 0.01; Fig. 7a and c). By contrast, the expression levels of N-cadherin and Ki-67 in 5-Fu group and PBMF-treated groups were significantly down-regulated compared with those in the model control group (*p* < 0.01; Fig. 7a, d and e).

## Discussion

Gastric cancer, one of the most common gastrointestinal malignancies worldwide, poses a severe threat on human health because of its “three high and three low” characteristics, namely, high incidence, metastasis, and mortality rates as well as the low early diagnosis, radical resection, and 5-year survival rates [3]. The prognosis of patients with gastric cancer is severely hampered by the limitation of surgical resection, serious side effects, sustained emergence of drug-resistance, and other adverse factors. Previous studies reported that a variety of phytochemicals, bioactive components, or secondary metabolites derived from natural plants possess favorable anti-tumor effects against malignant solid cancers; these compounds also have numerous advantages of less side effects, cost effectiveness, accessibility and so on and thus might be used as a novel complementary and alternative therapeutic strategy [26–28]. In theory, consumption of whole plant-based functional foods, which are superior natural resources of diverse phytochemicals, could exert additive or synergistic effects against cancer in comparison with an isolated agent [17]. In the present study, PBMF is composed of mixed phytochemical extracts mainly from *Coix* seed, *L. edodes*, *A. officinalis L.*, *H. cordata*, *Dandelion*, and *G. frondosa*, in accordance with the aforementioned theory. In recent years, phytochemicals isolated from these medicinal and edible plants possess potential in suppressing malignances either in vitro or in vivo [9–14]. Nevertheless, the combined inhibition effects of these natural plants on gastric carcinoma remain unknown and should be further investigated.

In this study, a subcutaneous xenograft tumor model from SGC-7901 cells was successfully established to investigate the effects of PBMF on tumor growth and elucidate the potential molecular mechanisms of action. The daily food intake of mouse is 8 g/100 g of its body weight [29]. In this regard, specific diets in low-, medium-, and high-dose groups were formulated with PBMF at concentrations of 43.22, 86.44 and 172.88 g/kg, respectively, which are equivalent to 5-, 10-, and 20-fold of the recommended intake for normal adults. 5-Fu was selected as the positive control agent because of its wide utilization in clinical chemotherapeutic treatment for gastrointestinal malignances [30]. After dietary administration with PBMF for 30 days continuously, the volume and weight of xenograft tumors resected from nude mice were lower than those in the model control group, as predicted. Hence, PBMF effectively inhibited xenograft tumor growth.

The results of H&E and TUNEL staining showed that PBMF significantly induced tumor necrosis and apoptosis. Subsequent ELISA analysis indicated that PBMF evidently diminished the serum level of TNF-α, which is a common anti-neoplastic cytokine that can induce directly the necrosis of cancer cells [31]. Nevertheless, the underlying molecular mechanisms remain elusive.

As a natural defense mechanism in an organism, apoptosis is responsible for elimination of unwanted cells and maintenance of homeostasis. With the initiation of apoptotic defects, the abnormal accumulation of unwanted cells eventually contributes to carcinogenesis [32]. Cancer cells utilize various strategies to evade apoptosis, where dysregulation of intrinsic and extrinsic apoptotic pathways remains indispensably orchestrated by enormous family of genes [33]. The critical regulator and executor of the intrinsic apoptotic pathway is the Bcl-2 family of genes, which are divided into anti-apoptotic (Bcl-2, Bcl-X, and Bcl-XL) or pro-apoptotic (Bax, Bak, and Bad) subfamilies [34, 35]. Bax/Bcl-2 ratio, which indicates the balance between anti-apoptotic and pro-apoptotic genes, directly determines cell fate between survival and death [36]. Moreover, caspase family members are equally essential to the intrinsic apoptotic pathway [37]. In early apoptosis, up-regulation of Bax can stimulate the formation of apoptosome complex via release of cytochrome c into cytosol, which then activates caspase-9 as initiator prior to cleaved activation of caspase-3 as effector [38–40]. Samana et al. [10] and Jinjuan et al. [12] reported that *L. edodes* and *H. cordata* extracts can significantly induce the apoptosis of SGC-7901 cells via up-regulation of Bax/Bcl-2 ratio at protein levels and activation of caspase-3. In the present study, we found the significant down-regulation of *Bcl-2* expression and the significant up-regulation of *Bax*, *Caspase-3*, and *Caspase-9* and increase in Bax/Bcl-2 ratio following PBMF treatment. This finding indicates that the pro-apoptosis effect depends on breaking the equilibrium of Bcl-2 family members and in turn activating caspase cascade reaction.

Metastasis, an important biological characteristic of cancers, plays a vital role in cancer progression and accounts for approximately 90% of cancer-related mortality [41]. EMT is a sophisticated and multi-step cellular phenotypic transition from stationary epithelial cells into highly migratory and invasive mesenchymal cells, which can promote cancer metastasis [42]. In the above process, the high expression of E-cadherin, as a crucial epithelial marker, benefits the maintenance of epithelial integrity against migration, whereas the high expression of necessary mesenchymal markers, such as N-cadherin and vimentin, facilitate the acquisition of invasive and migratory properties [43]. Furthermore, transcription factors, including Snail (Snail 1), Slug (Snail 2), Twist, MMPs, and lymphoid enhancer binding factor-1 (LEF-1) can repress the expression of E-cadherin directly or indirectly and then trigger an EMT event [44]. Rilei et al. declared that sodium new houttuyfonate (SNH), a main ingredient of *H. cordata*, can regress the EMT progression by augmenting E-cadherin protein level and diminishing N-cadherin protein level, thereby effectively alleviating metastasis in NSCLC cell [45]. In the present study, we found notable increase in the mRNA expression of *E-cadherin* and decrease in the mRNA expression of *N-cadherin* and *Snail* with treatment of PBMF, consistent with their counterparts at the protein level. Hence, PBMF seems to reverse EMT and in turn exerts anti-metastasis property.

Matrix metalloproteinases (MMPs), a family of zinc-dependent endopeptidases, degrade the extracellular matrix and facilitate the metastasis of malignances [46]. A previous research confirmed the robust correlation between high MMP-2/9 expression and metastasis in human gastric cancer; hence, their overexpression could be a valuable predictor of poor prognosis in patients with gastric cancer [47, 48]. Samana et al. showed that *L. edodes* extract can attenuate the protein levels of MMP-2 and MMP-9 to suppress the migration and invasive potential of SGC-7901 cells [10]. The current experimental data demonstrated that PBMF obviously decreased the expression levels of MMP-2 and MMP-9 at serum and mRNA levels. Hence, the anti-metastasis effect of PBMF might also involve the negative regulation of MMP-2/9.

The canonical Wnt/β-catenin signaling pathway plays a pivotal role in the proliferation, apoptosis, invasion, and migration of malignant tumors [49, 50]. This pathway is closely associated with the development of massive human cancers, including gastric cancer. Approximately 40% of patients with gastric cancer have dysfunction in the Wnt/β-catenin signaling pathway [51]. When Wnt signals are absent, the majority of β-catenin located at the cytoplasmic side of the membrane combines with E-cadherin and then generates the β-catenin/E-cadherin complex, which stabilizes the cell adhesion. The rest of the dissociated β-catenin in the cytoplasm initiates phosphorylated degradation by the multi-protein destruction complex composed of axis inhibition protein (AXIN), adenomatous polyposis coli (APC), and glycogen synthase kinase-3β (GSK-3β). Accordingly, the cytoplasmic levels of free β-catenin are normally maintained at minimum. By contrast, when the Wnt ligands bind to Frizzled (FZ) receptors and low-density lipoprotein receptor-related protein 5/6 (LRP5/6) coreceptors, Dishevelled (Dvl or Dsh) protein is recruited to promote the phosphorylation of GSK-3β and in turn destabilize the β-catenin destruction complex, thereby hindering the degradation of β-catenin. β-catenin that aberrantly accumulates in the cytoplasm is allowed to be translocated into the nucleus and interacts with T-cell factor/Lymphoid enhance factor (TCF/LEF) transcription factors; this process is accompanied by the activation of downstream target genes, such as Bcl-2, Cyclin D1, C-myc, MMPs, N-cadhein, and Snail [52–54]. Overall, changes in the stability of β-catenin decided by GSK-3β must be key to regulate this signaling pathway. Jiabing et al. demonstrated that Qiyusanlong decoction composed of 10 kinds of traditional Chinese herbs, including *Coix* seed, can suppress lung carcinoma development by negative modulation of the Wnt/β-catenin signaling pathway [55]. In the current study, PBMF treatment remarkably decreased β-catenin expression but increased GSK-3β expression at mRNA and/or protein levels, thereby hindering the Wnt/β-catenin signaling pathway. As such, the effects of PBMF on pro-apoptosis and EMT reversal are rationally related to the regulation of specific downstream factors via the Wnt/β-catenin signaling pathway.

Cyclin D1, as one of numerous downstream genes in this signaling pathway, regulates the G1/S checkpoint of cell cycle progression; overexpression of this gene can induce cell cycle arrest and ultimately promote cell proliferation. Meanwhile, the dysregulation of Cyclin D1 prevalently occurs in the majority of human cancers and is closely linked to shorter survival of patients [56–58]. In addition, the high expression of the Ki-67 protein, a recognized cell proliferation marker, is widely used as positive indicator of poor prognosis in many cancers [59, 60]. Fun et al. confirmed that a steroidal saponin from *A. officinalis L.* can induce the G0/1 cell cycle arrest by down-regulating the Cyclin D1 protein in the Ishikawa endometrial cancer cell, suggesting the anti-proliferative efficacy of the extract [11]. In the present study, we found that the protein expression of Ki-67 and the mRNA expression of *Cyclin D1* were significantly reduced after PBMF treatment, demonstrating that the anti-proliferation effect is associated with the regulation of Cyclin D1. Moreover, existing data indicate that PBMF could down-regulate CyclinD1 expression by suppressing the Wnt/β-catenin signaling pathway.

## Conclusions

PBMF tested in a nude mouse xenograft model from SGC-7901 cells exhibited strong anti-tumor effects against gastric cancer by inhibiting tumor growth and tumor cell proliferation and inducing apoptosis. The underlying mechanisms primarily rely on EMT reversal and transcriptional inactivation of key downstream target factors by blocking the Wnt/β-catenin signaling pathway. In general, the present study provides insights into the application of PBMF in complementary and alternative therapies for gastric cancer.

## Supplementary Information

**Additional file 1.**

**Additional file 2.**

**Additional file 3.**

**Additional file 4.**

**Additional file 5.**

**Additional file 6.**

**Additional file 7.**

## Data Availability

The data and materials supporting the conclusions of this study are manifested and described within this manuscript.

## Abbreviations

PBMF: Plant-based medicinal food
EMT: Epithelial-mesenchymal transition
H&E staining: Hematoxylin and eosin staining
TUNEL staining: Terminal deoxynucleotidyl transferase-mediated dUTP nick end labeling staining
ELISA: Enzyme-linked immunosorbent assay
RT-qPCR: Real-time quantitative Polymerase Chain Reaction
5-Fu: 5-Fluorouracil
PBS: Phosphate buffered saline
TNF-α: Tumor necrosis factor-α
MMP-2/9: Matrix metalloproteinase-2/9
GSK-3β: Glycogen synthase kinase-3β
AXIN: Axis inhibition protein
APC: Adenomatous polyposis coli
FZ: Frizzled
Dvl/Dsh: Dishevelled
LRP-5/6: Low-density lipoprotein receptor-related protein 5/6
TCF/LEF: T-cell factor/Lymphoid enhance factor
